# Methotrexate Scarcity Among Children’s Oncology Group Institutions: Results of a Multinational Survey

**DOI:** 10.1093/oncolo/oyad323

**Published:** 2023-12-09

**Authors:** Sarah Menig, Olga Militano, Andrew Ostrenga, M Brooke Bernhardt, Brittany Lee, Yoram Unguru

**Affiliations:** Department of Pharmacy, Seattle Children’s Hospital, Seattle, WA, USA; Children’s Oncology Group, Monrovia, CA, USA; Department of Pharmacy, University of Mississippi Medical Center, Children’s of Mississippi, Jackson, MS, USA; Division of Pharmaceutical Services, Department of Pharmacy and Pharmaceutical Sciences, St. Jude Children’s Research Hospital, Memphis, TN, USA; Ben Towne Center for Childhood Cancer Research, Seattle Children’s Research Institute, Seattle, WA, USA; Department of Pediatrics, University of Washington, Seattle, WA, USA; Division of Pediatric Hematology/Oncology, The Herman and Walter Samuelson Children’s Hospital at Sinai, Baltimore, MD, USA; Johns Hopkins Berman Institute of Bioethics, Baltimore, MD, USA

**Keywords:** chemotherapy, methotrexate, drug shortages, mitigation strategies, equity, access, pediatric oncology

## Abstract

Shortages of curative chemotherapy agents for children and adults with cancer are ubiquitous. These shortages directly result in compromised outcomes, increased medication errors, heightened cost to health systems, and patient deaths. Methotrexate is a staple of many curative childhood cancer regimens and is frequently in scarcity. No national guidance to manage methotrexate and other chemotherapy shortages exists. To assess the effect of the current methotrexate shortage, a multinational survey of Children’s Oncology Group (COG) member institutions was conducted. Wide variation in the scope of methotrexate shortage in the US was demonstrated; some centers experienced significant scarcity while others experienced no shortage. Methotrexate mitigation strategies differed by COG site, resulting in potential to exacerbate differential access to life-saving medication and inequities in care. Preventing chemotherapy shortages remains a challenge. In the interim, standard guidance to assist clinicians to equitably and fairly cope with methotrexate and related drug shortages is needed.

**Implications for Practice**

Oncology teams in general, and pediatric oncology teams in particular, frequently face drug shortages. Methotrexate is a standard chemotherapeutic used as part of curative regimens for a range of childhood cancers. Methotrexate has frequently been in short supply, yet national guidance to manage methotrexate and other chemotherapy shortages is lacking. This multinational study found wide variation in the scope of methotrexate shortage with some centers experiencing significant scarcity while others experienced no such shortage. Moreover, methotrexate mitigation strategies differ among pediatric oncology clinicians, resulting in potential to exacerbate differential access to life-saving medication and inequities in care. Policymakers and industry experts may benefit from these findings as they attempt to meaningfully address drug shortages.

## Introduction

The current methotrexate injection shortage is one of the most persistent and potentially impactful US chemotherapy shortages. Initially reported in early February 2023 by the American Society of Health-Systems Pharmacists (ASHP),^1^ the methotrexate shortage is estimated to continue through the fourth quarter of 2023 or beyond. Like other shortages of sterile generic injectable chemotherapeutics (SGIC), methotrexate injection shortage is cyclical, with prior shortages reported in 2012 and 2016.^1,2^

The primary cause for the methotrexate shortage is a voluntary withdrawal of injectable methotrexate from the US market by Intas Pharmaceuticals,^3^ which manufactured several generic chemotherapy products for its US distributor Accord Pharma, including methotrexate, cisplatin, and carboplatin. Prior to this voluntary withdrawal, Accord Pharma held 33% of the US market share of injectable methotrexate.^4^ Egregious quality control issues identified during a Food and Drug Administration (FDA) inspection^5^ of the Intas manufacturing facility in the Special Economic Zone near Ahmedabad, India led to the voluntary product withdrawal. Quality control issues identified at the facility included, but were not limited to, discarded, missing, and purposefully destroyed current good manufacturing practice records and failure to abide by a range of quality assurance standards in the drug production process. Furthermore, the worldwide COVID-19 pandemic negatively affected the FDA’s ability to inspect medication manufacturing facilities outside of the US with the number of 2021 foreign inspections dropping to <15% of pre-pandemic numbers,^6,7^ potentially contributing to some of the discovered quality control issues. It is well-recognized that the complexity in manufacturing SGIC, as well as company-specific business decisions, contributes to drug shortages and, indeed, the remaining 4 US methotrexate manufacturers (Pfizer, Hikma, Fresenius Kabi, and Teva) continue to struggle to fill the market void caused by the Accord product withdrawal. As reported by the FDA, “drug manufacturers make business decisions across the life cycle of a drug.”^8^ These decisions are influenced by a range of factors, including a drug’s decreasing profitability, which as recognized by the FDA and others, may result in a manufacturer discontinuing production resulting in, or contributing to, existing drug scarcity. Drug shortages are compounded when manufacturers approved to market scarce drugs, consciously decide against doing so due to business decisions.^8-10^

The methotrexate shortage disproportionally affects pediatric oncology patients. Methotrexate is a cornerstone of standard of care and curative regimens for a wide variety of pediatric malignancies, including acute lymphoblastic leukemia (ALL), non-Hodgkin lymphoma, central nervous system tumors, and osteosarcoma, accounting for the majority of all childhood patients with cancer. In most cases, there is no substitute or alternative medication for methotrexate and those with alternative treatment regimens lack robust outcome data. In addition to clinical care, shortages of chemotherapy such as methotrexate, also negatively affect clinical trial enrollment,^11^ limiting the ability to better understand disease processes and improve patient outcomes. Specifically, the methotrexate shortage has affected 8 open Children’s Oncology Group (COG) trials, of which 5 are trials for ALL (the most common pediatric malignancy). Beginning in February 2023, with an update in May 2023, COG leadership posted Methotrexate Shortage Memos for its members with recommendations for management of patients enrolled on COG clinical trials affected by the shortage.

As a result of the long-standing injectable methotrexate shortage, the COG Pharmacy Steering Committee conducted a survey of COG member institutions to assess the impact of the shortage on patient care, trial enrollment, and mitigation strategies employed.

## Methods

A 14-item survey was developed by the COG Pharmacy Steering Committee and distributed via email by the COG Communications Department (Supplementary Fig. S1). Survey data were collected and managed using REDCap electronic data capture tools hosted at Seattle Children’s Hospital.^12,13^ Survey items were formulated based upon expert opinion of COG Pharmacy Committee members who have extensive experience with chemotherapy shortages. Pharmacy professionals caring for pediatric oncology patients were the primary target of the survey. The survey was sent to each COG site Responsible Investigator Pharmacist (RI Pharm) or another designated pharmacist (in cases where an RI Pharm was not listed) at 227 COG member institutions across 5 countries (US, Canada, Saudi Arabia, Australia, and New Zealand). An RI Pharm designation identified a pharmacist responsible for overseeing COG Pharmacy practices at a particular institution. The email survey invitation described the purpose of the survey and provided a web link to the survey. The invitation stated that anonymized data would be shared within COG for educational purposes, including methods for managing the current methotrexate injection shortage, and that it was not related to the COG Quality Assurance/Audit team activity. Data collection began on July 14, 2023 and closed on August 4, 2023.

Data collection included facility name, country, and US geographic region. Survey items queried the impact of methotrexate shortage on patient care, clinical trial enrollment, implemented mitigation strategies, changes in drug procurement and patient referral patterns, as well as potential future mitigation strategies (Supplementary Fig. S1). Survey responses were de-identified. This analysis of the existing data set initially obtained for public health purposes was determined by Seattle Children’s Hospital to be exempt from Institutional Review Board review. Descriptive statistics were used to describe the frequency of responses to each survey question and chi-square test was used to compare regional differences in impact and percent of sites that had implemented mitigation strategies.

## Results

Seventy responses (67 from US sites) were received for a response rate of 31% (Table 1). Two responders from Canadian institutions and one from an Australian institution stated that their countries were not affected by the methotrexate shortage.

**Table 1.** Survey respondent geographic location (*n* = 70)

Location	Number of respondents (%)
**US**	**67 (96)**
Northeast	17 (25)
Southeast	14 (21)
Midwest	14 (21)
West	12 (18)
Southeast	10 (15)
**Canada**	**2 (3)**
**Australia**	**1 (1)**

**Figure 1.**
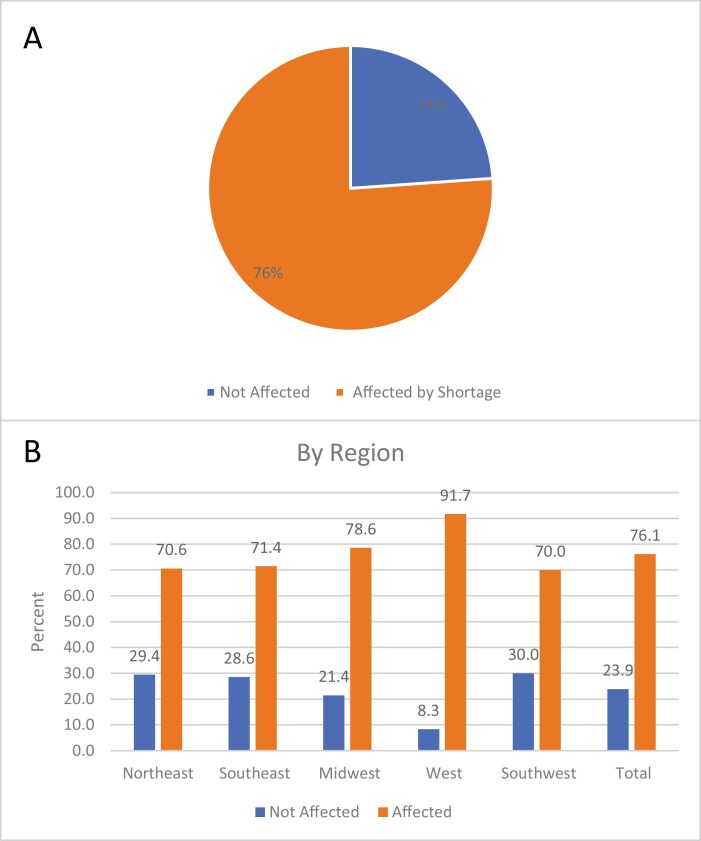
Methotrexate shortage by COG site and region. Percent of US respondent sites affected by the methotrexate shortage, (**A**) all US respondents, (**B**) by region. Differences in impact by region were not statistically significant (*P* = .68).

Of the 67 US-based respondents representing individual pediatric oncology centers, 76% (*n* = 51) indicated that they were affected by the methotrexate shortage (Fig. 1). No statistically significant differences of the methotrexate injectable shortage occurred by region (Northeast, Southeast, West, Midwest, Southwest US, *P* = .68), although institutions in the West region appeared to have numerically higher percentage of affected sites (91.7%, *n* = 11). Nine percent of sites (*n* = 6) endorsed that the methotrexate shortage precluded patient enrollment on clinical trials.

**Figure 2.**
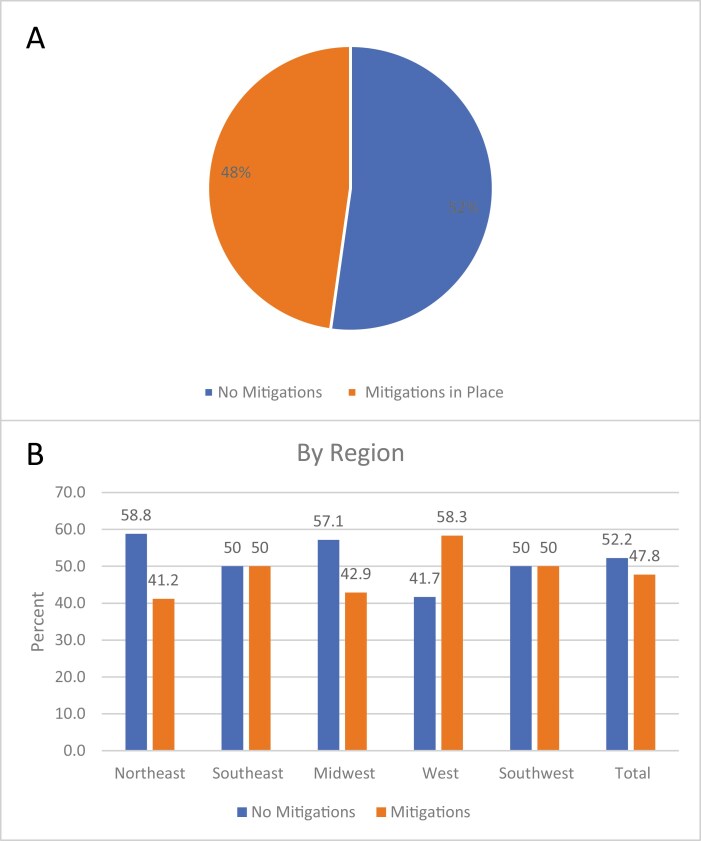
Methotrexate shortage mitigation by COG site and region. Percent of US respondent sites who have implemented mitigation strategies as a result of the methotrexate shortage, (**A**) all US respondents, (**B**) by region. Differences in need for implementation of mitigation by region were not statistically significant (*P* = .91).

**Figure 3.**
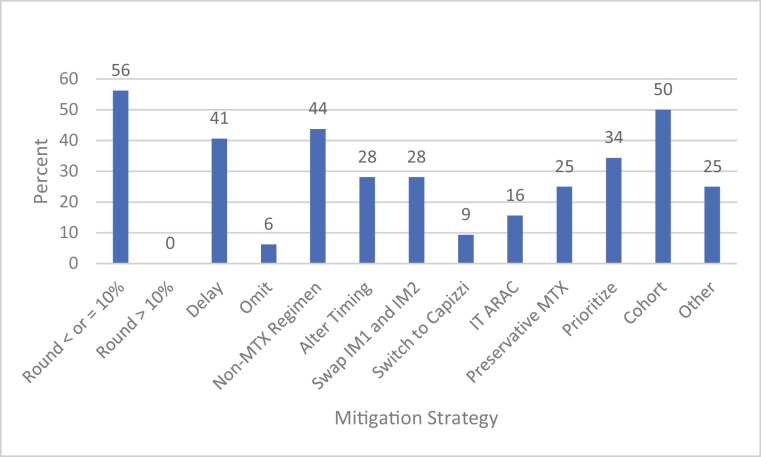
Methotrexate mitigation strategy. Abbreviations: Capizzi, escalating dose methotrexate with asparaginase; IM1, interim maintenance 1; IM2, interim maintenance 2; IT ARAC, intrathecal cytarabine; MTX, methotrexate.

**Figure 4.**
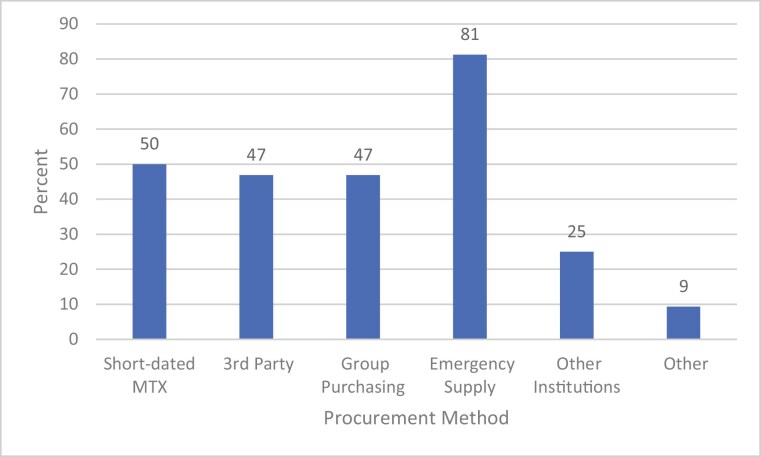
Methotrexate procurement method. Abbreviation: MTX, methotrexate.

As a result of the shortage, 48% of respondents (*n* = 32) implemented methotrexate mitigation strategies at their institutions (Fig. 2). There were no statistically significant regional differences in frequency of centers implementing mitigation strategies (*P* = .91). The most implemented mitigation strategies included methotrexate dose rounding by ≤10% (56% of respondents, *n* = 18), cohorting patients to minimize single vial waste (50%, *n* = 16), selecting a non-methotrexate–containing regimen (44%, *n* = 14), and delaying therapy (41%, *n* = 13). An additional 34% (*n* = 11) of centers prioritized patients by age or disease (Fig. 3); data were not collected on prioritization methods. Of the 32 sites implementing methotrexate shortage mitigation, the majority had to procure emergency supply from manufacturers (81%, *n* = 26), half of sites (*n* = 16) procured short-dated methotrexate (product with an impending expiration date), and 47% (*n* = 15) procured methotrexate from third-party distributors at a higher cost (Fig. 4). Of the 32 institutions with mitigations in place, 15.6% (*n* = 5) were unable to accept patients referred to their institution, and 9.4% (*n* = 3) transferred patients to a different facility due to this shortage.

All respondents were queried about potential future mitigations they might consider. Responses are available for all US and Canadian respondents (*n* = 69). The most reported mitigation strategies for future consideration included altering the timing of methotrexate-containing cycles (62%, *n* = 43), dose rounding by ≤10% (59%, *n* = 41), and delaying therapy (48%, *n* = 33). Forty-five percent of respondents also reported they would consider using preservative-containing methotrexate when appropriate, 43% would consider prioritizing patients, and 42% would consider cohorting patients (Supplementary Fig. S2 shows potential future mitigation strategies). Most respondents (>50%) reported they would consider each of the listed procurement strategies. The most highly considered procurement strategy was obtaining emergency supply from manufacturers 84% (Supplementary Fig. S3 shows potential future procurement methods). Forty-six percent and 43%, respectively, reported their center would consider refusing patient referral or transfer patients to a different facility related to the methotrexate shortage.

## Discussion

Shortages of drugs critical to childhood cancer treatment are no longer the new normal, but simply, normal. Patients, pediatric oncology professionals, and health systems are repeatedly challenged by the myriad of shortage-related problems. While existing ethical frameworks and allocation guidelines provide helpful guidance to pediatric oncology teams,^14,15^ the lack of a standardized national approach has resulted in wide variation across (and potentially within) institutions, often in the absence of evidence. This may explain the variety of mitigation strategies utilized (or considered) by institutions represented in this cohort.

While there were no statistically significant regional differences in methotrexate injectable shortage impact among institutions within the US, the findings of this survey also highlight wide inequities in drug access among centers. A quarter of respondents did not experience a methotrexate shortage, while others were so affected, they were unable to accept a referred patient or were forced to transfer a patient to another center. Future study of additional institutional factors—such as institution size, affiliation with an adult oncology program, dedicated purchasing pharmacist role, or institution membership in particular group purchasing organizations—is warranted to identify factors that may impact this differential access to a drug in short supply. Greater transparency regarding drug distribution from manufacturers and wholesalers during times of shortage is also imperative to help address inequities in access. Just under half of sites (47%) who had implemented mitigations due to the methotrexate shortage sourced medication from third-party distributors at a higher cost and 77% of respondents said their site would consider this procurement strategy, underscoring the financial impact of drug shortages and its potential to exacerbate inequities. Furthermore, knowing what process existed at individual centers for making drug allocation decisions is essential. Were decisions based upon published recommendations? Was a drug shortage committee or similar body involved and if so, what was the makeup of this body? How, and to whom was information about methotrexate shortages disseminated? Were patients and families informed of the shortage and allocation decision-making process? Was an appeals process for challenging prioritization decisions in place? Were specific measures considered or enforced to limit existing inequities in care?

Arguably, the primary study limitation is the low response rate. Given time urgency and our effort to promote heightened awareness at the peak of the methotrexate shortage, we purposefully opted not to extend the survey window, nor did we formally validate the survey prior to its distribution. We also acknowledge the understandably vague survey question regarding potential future mitigation strategies may have influenced how respondents interpreted this question. Originally designed for public health purposes, our intention was to provide information on possible mitigation strategies for those not yet affected and possible next steps for centers to consider when existing mitigation strategies are inadequate. Individual responses are likely dependent upon the severity of the shortage experienced at each institution at the time of survey completion. Future study to elucidate standardized, stepwise approaches to mitigation along with clinical outcome data on the safety and effectiveness of each mitigation strategy as well as the associated cost of such measures is needed to ensure more equitable care in future shortages. While the limited response rate in this report precluded doing so, categorizing strategies by region in future study would be helpful in explaining any regional differences in outcomes. Lastly, qualitative data arguably would have enriched our sample. Limitations notwithstanding, our findings shed light on a previously unstudied topic with broad implications beyond childhood cancer.

As we confront yet another significant drug shortage, we must reflect on the lack of progress. Two of the authors of this report are members of a taskforce that previously published an allocation framework to assist pediatric oncology clinicians in prioritizing scarce chemotherapy and supportive care agents.^14,15^ At the time of publication more than 7 years ago, the authors called for a national policy advisory statement for allocating scarce chemotherapy along with meaningful engagement by policymakers with stakeholders to prevent shortages once and for all. Unfortunately, neither of these steps has occurred. The lack of meaningful reform has resulted in unnecessary suffering by vulnerable children with cancer and their families, significant cost to health systems, and moral injury experienced by countless pediatric oncology professionals. Furthermore, the mitigation strategies many centers currently implement may potentially exacerbate existing inequities in care and access to lifesaving medications. Academics and professional groups have proposed thoughtful solutions to the drug shortage problem and governmental bodies have convened.^8,14-18^ Recommendations include, but are not limited to, private-public partnerships, offering pharmaceutical manufacturers incentives and subsidies while rewarding manufacturers for maintaining ­quality metrics, establishing a single information source for shortage-related information allowing for early and consistent notification, requiring pharmaceutical companies to disclose reasons for disrupted supply (as called for by the CARES Act), and pivoting from the existing just-in-time production model allowing for more drug readily available and on pharmacy shelves. Too often, children with cancer are overlooked. For example, a recent report of 7 scarce chemotherapy agents, including methotrexate, cisplatin, and carboplatin, focuses exclusively on adult cancer, failing to mention a single pediatric oncology indication^4^; each of these 3 drugs is utilized across childhood cancer. Notably, during the writing of this manuscript, 2 additional chemotherapy agents, dacarbazine and vinblastine, were added to the growing chemotherapy shortage list. Like methotrexate and cisplatin, which comprise two-thirds of curative regimens for osteosarcoma, dacarbazine and vinblastine are standard elements of curative regimens for Hodgkin lymphoma, which affects even more children, adolescents, and young adults than osteosarcoma. Urgent legislation is critical to prevent further shortages and patient harm to children with cancer.

## Supplementary Material

oyad323_suppl_Supplementary_Figures_1

oyad323_suppl_Supplementary_Figures_2

oyad323_suppl_Supplementary_Figures_3

## Data Availability

The data underlying this article are available in the article and in its online supplementary material.
