# Supplementary material for: Methotrexate Scarcity Among Children’s Oncology Group Institutions: Results of a Multinational Survey
Source: Oncologist. 2023 Dec 9;30(8):oyad323. doi: 10.1093/oncolo/oyad323 (PMC12396948; doi:10.1093/oncolo/oyad323)
Supplement: oyad323_suppl_Supplementary_Figures_1 [file oyad323_suppl_supplementary_figures_1.pdf]

Methotrexate Shortage Survey

The following survey is not related to the COG QA/Audit team and survey responses are intended for information collection and future planning only. Measures listed below are not intended as formal study recommendations. Study specific recommendations can be found on the main COG protocols webpage under the drug shortages tab, or the individual protocol page.

Current Impact of Methotrexate Shortage

1 Has your institution been affected by the methotrexate shortage?

- ◯ Yes
- ◯ No

2 Did the methotrexate shortage result in inability to enroll patients on clinical trials?

- ◯ Yes
- ◯ No

3 Has your institution altered patient's therapy or implemented mitigation strategies due to the methotrexate shortage?

- ◯ Yes
- ◯ No

Current Mitigation Strategies

4 What treatment-related measures has your institution implemented due to the methotrexate shortage (check all that apply):

- ☐ Reducing methotrexate dose by LESS THAN or equal to 10%
- ☐ Reducing methotrexate dose by GREATER THAN 10%
- ☐ Delaying methotrexate therapy cycles/doses
- ☐ Omitting methotrexate doses (without substitution)
- ☐ Changing therapy to a non-methotrexate containing regimen
- ☐ Alter timing of methotrexate therapy cycles with non-methotrexate containing cycles with hope of later sufficient availability of preservative-free methotrexate
- ☐ For acute lymphoblastic leukemia (ALL) and acute lymphoblastic lymphoma (LLy) patients: switching high dose methotrexate cycles with Capizzi methotrexate (escalating dose with asparaginase) cycles with hope of later availability of preservative-free methotrexate
- ☐ For ALL and LLy patients: changing high dose methotrexate cycles to Capizzi methotrexate (escalating dose with asparaginase) cycles so the patient receives two Capizzi courses
- ☐ For ALL, acute myeloid leukemia (AML), LLy patients: switching intrathecal (IT) methotrexate to IT cytarabine
- ☐ Use of methotrexate containing preservatives for the first 30 minute infusion of HIGH dose or intermediate dose methotrexate
- ☐ Prioritizing patients by age or disease
- ☐ Cohorting patients
- ☐ Other measures (please specify)
- ☐ None

Other measures (please specify) ______________________________

5 What procurement-related measures has your institution implemented due to the methotrexate shortage (check all that apply):

- ☐ Procuring short-dated methotrexate
- ☐ Procuring methotrexate from Third Party Distributors at a higher cost
- ☐ Procuring methotrexate from Group Purchasing Organization (GPO) strategic supply
- ☐ Procuring methotrexate emergency supply directly from manufacturers
- ☐ Receiving methotrexate from other organizations
- ☐ Other measures (please specify)
- ☐ None

Other measures (please specify): __________________________________

6 Has your institution refused patient referrals from outside institutions due to the methotrexate shortage?

- ◯ Yes
- ◯ No

7 Has your institution referred patients to other institutions (smaller practices or clinics closer to home) as a result of the methotrexate shortage?

- ◯ Yes
- ◯ No

Please share any additional comments: __________________________________

Future mitigation strategies considered

1 What treatment-related measures would your institution consider implementing due to the methotrexate shortage (check all that apply):

- ☐ Reducing methotrexate dose by LESS THAN or equal to 10%
- ☐ Reducing methotrexate dose by GREATER THAN 10%
- ☐ Delaying methotrexate therapy cycles/doses
- ☐ Omitting methotrexate doses (without substitution)
- ☐ Changing therapy to a non-methotrexate containing regimen
- ☐ Alter timing of methotrexate therapy cycles with non-methotrexate cycles with hope of later sufficient availability of preservative-free methotrexate
- ☐ For ALL, LLy patients: switching high dose methotrexate cycles with Capizzi methotrexate (escalating dose with asparaginase) cycles with hope of later availability
- ☐ For ALL, LLy patients: changing high dose methotrexate cycles to Capizzi methotrexate (escalating dose with asparaginase) cycles so the patient receives two Capizzi courses
- ☐ For ALL, AML, LLy patients: switching intrathecal (IT) methotrexate to IT cytarabine
- ☐ Use of methotrexate containing preservatives for the first 30 minute infusion of HIGH dose or intermediate dose methotrexate
- ☐ Prioritizing patients by age or disease
- ☐ Cohorting patients
- ☐ Other measures (please specify)
- ☐ None

Other measures (please specify): __________________________________

2 What procurement-related measures would your institution consider implementing due to the methotrexate shortage (check all that apply):

- ☐ Procuring short-dated methotrexate
- ☐ Procuring methotrexate from Third Party Distributors at a higher cost
- ☐ Procuring methotrexate from Group Purchasing Organization (GPO) strategic supply
- ☐ Procuring methotrexate from other organizations
- ☐ Procuring methotrexate emergency supply directly from manufacturers
- ☐ other measures (please specify)
- ☐ None

Other measures (please specify): __________________________________

3 Would your institution consider refusing patient referral from outside institutions as a result of the methotrexate shortage?

- ◯ Yes
- ◯ No

4 Would your institution consider referring patients to other institutions (smaller practices or clinics closer to home) as a result of the methotrexate shortage?

- ◯ Yes
- ◯ No

Please share any additional comments __________________________________

Demographic Information

Please list your institution (this information is ONLY being collected to prevent duplicate data, results will remain anonymous). __________________________________

What country is your institution located?

- ◯ USA
- ◯ Canada
- ◯ Australia
- ◯ New Zealand

If located in the US, in what region is your institution located? (see regional map at https://education.nationalgeographic.org/resource/united-states-regions/)

- ◯ Northeast
- ◯ Southeast
- ◯ Midwest
- ◯ West
- ◯ Southwest
