# Supplementary figures and images for: Methotrexate Scarcity Among Children’s Oncology Group Institutions: Results of a Multinational Survey
Source: Oncologist. 2023 Dec 9;30(8):oyad323. doi: 10.1093/oncolo/oyad323 (PMC12396948; doi:10.1093/oncolo/oyad323)

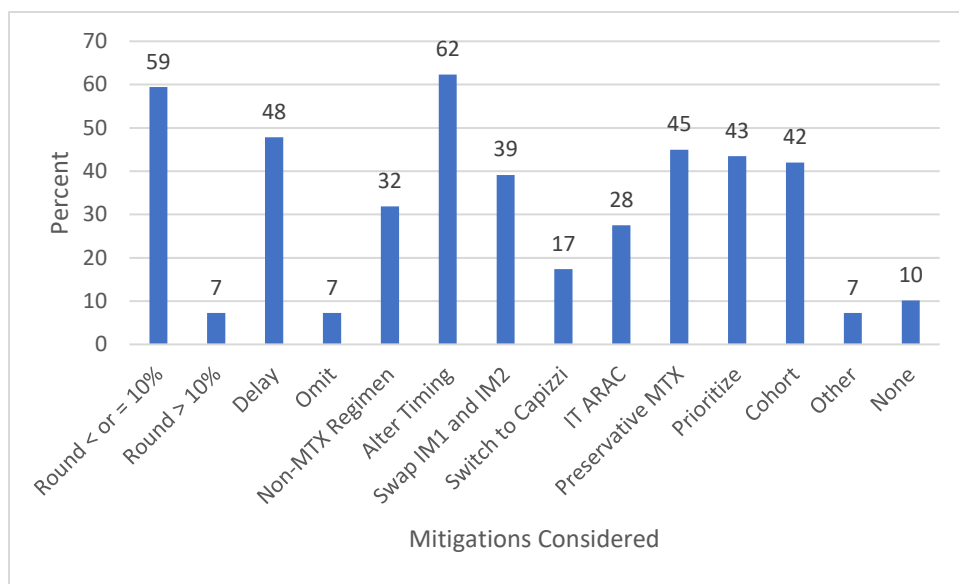

Supplement: oyad323_suppl_Supplementary_Figures_2 [file oyad323_suppl_supplementary_figures_2.pdf]

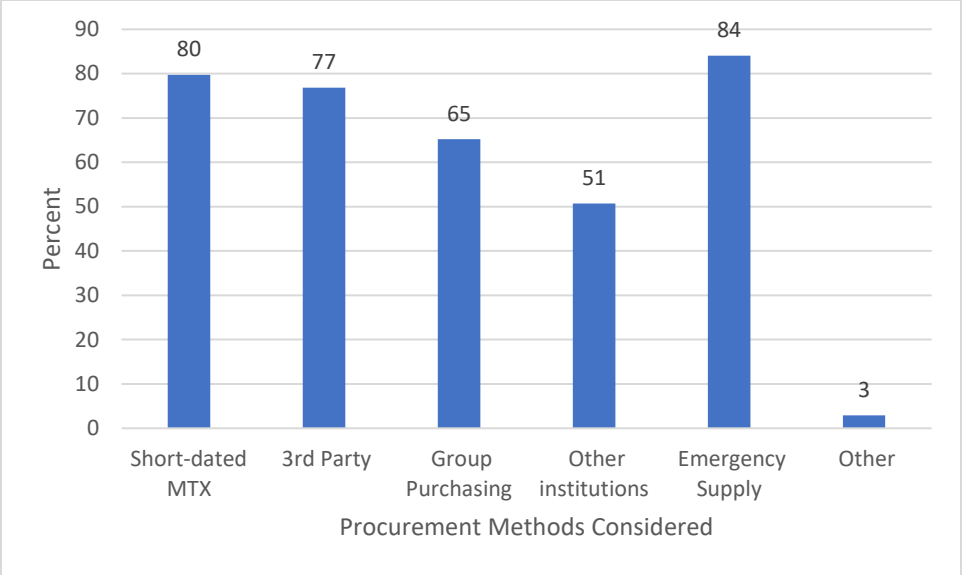

Supplement: oyad323_suppl_Supplementary_Figures_3 [file oyad323_suppl_supplementary_figures_3.pdf]
